# Supplementary material for: Polyketide synthase gene clusters in the ericoid mycorrhizal fungus Oidiodendron maius: genome-wide prediction, expression profiling, and functional characterization of knockout mutants
Source: Front Plant Sci. 2026 Sep 16;17:1924430. doi: 10.3389/fpls.2026.1924430 (PMC13623903; doi:10.3389/fpls.2026.1924430)
Supplement: Supplementary file 1 [file SupplementaryFile1.zip › Supplementary Materials/Supplementary Folder 1.zip!/Oidma1_AssemblyScaffolds_Repeatmasked/knownclusterblast/cluster42/jgi.p_Oidma1_171090_mibig_hits.html]

| MIBiG Protein | Description | MIBiG Cluster | MiBiG Product | % ID | % Coverage | BLAST Score | E-value |
| --- | --- | --- | --- | --- | --- | --- | --- |
| AGO59052.1 | PtaK | BGC0000121 | Pestheic\_acid\_biosynthetic\_gene\_cluster | 47 | 82.4027072758 | 451 | 1.9e-126 |
| EAL89346.1 | extracellular\_dihydrogeodin\_oxidase/laccase,\_putative | BGC0001403 | Trypacidin\_biosynthetic\_gene\_cluster | 33 | 94.4162436548 | 299 | 6.9e-81 |
| AGO59042.1 | PtaE | BGC0000121 | Pestheic\_acid\_biosynthetic\_gene\_cluster | 32 | 84.0947546531 | 252 | 1.3e-66 |
